# Digital distraction and adolescent development: a narrative review of smartphone use, cognitive effects, and policy interventions

**DOI:** 10.3389/fpsyg.2026.1832219

**Published:** 2026-09-08

**Authors:** Derin Colakoglu, Cynthia van Golen

**Affiliations:** 1 Union County Vocational Technical School, Scotch Plains, NJ, United States; 2 Department of Biological Sciences, Delaware State University, Dover, DE, United States

**Keywords:** academic performance, adolescents, attention, digital media, mental health, Mobile phone bans, screen time, sleep disruption

## Abstract

Smartphone use has become nearly universal among adolescents, raising concerns about its potential effects on attention, well-being, and learning. Over the past decade, rapid growth in smartphone ownership and social media engagement among youth has prompted increasing interest in how digital technologies influence cognitive and psychological development. At the same time, schools and youth organizations have begun implementing policies that restrict smartphone use in an effort to reduce distraction and improve student engagement. Despite these efforts, the broader impacts of smartphone use and restriction policies on adolescent development remain a topic of active debate. This integrative narrative review synthesizes current evidence on smartphone use among adolescents, focusing on three primary domains: psychological well-being, cognitive functioning and learning, and emerging policy responses such as school cellphone bans. Research across observational studies, experimental work, and systematic reviews suggests that excessive smartphone use is associated with modest but consistent relationships with sleep disruption, reduced attentional capacity, and increased internalizing symptoms among some adolescents. However, these effects are heterogeneous and appear to be moderated by factors including type of digital activity, timing of use, and individual vulnerability. Evidence examining the effects of school-based smartphone bans indicates improvements in classroom attention and engagement, although broader impacts on mental health and long-term digital habits remain unclear. Furthermore, adolescent views on smart phone bans are mixed, with students recognizing that there are positive and negative effects of both smartphones and bans. Together, these findings suggest that smartphone use represents a complex developmental exposure rather than a uniformly harmful or beneficial influence. Future research should examine how structured phone-free environments, including school and extracurricular settings, influence adolescents’ social interaction, digital behavior, and well-being over time.

## Introduction

Digital technologies are deeply integrated into the social and developmental environments of children and adolescents. Among these, smartphones are some of the most influential innovations of the past 20 years, fundamentally changing how people communicate, access information, and engage in social activities. Since their debut in the late 2000s, smartphone adoption has surged worldwide. In the United States alone, smartphone ownership among adults rose from about 35% in 2011 to 91% in 2024 (Pew Research Center, 2024). Similar patterns have been seen internationally, demonstrating the widespread incorporation of mobile technologies into daily life (Twenge et al., 2019; Paulus et al., 2023). Along with the rise of smartphones, mobile apps, especially social media platforms, have become vital parts of the digital landscape. These have enabled constant connectivity and ongoing streams of information and social engagement.

Importantly, the expansion of smartphone use has been especially notable among children and adolescents. Over the past decade, smartphone access among U.S. teenagers has grown from about 73% to 95% (Pew Research Center, 2022). Additionally, smartphone use is now appearing at increasingly younger ages. Recent estimates indicate that roughly 37% of children aged 9 to 11 now own or regularly use a smartphone (Auxier et al., 2020), while most adolescents report being online “almost constantly” (Pew Research Center, 2023). These changes have been accompanied by significant increases in time spent engaging with digital media. Surveys on youth media consumption show that adolescents spend several hours each day on screen-based technologies, with the most popular platforms including video-sharing services, messaging apps, and short-form social media sites like TikTok, Instagram, and Snapchat (Common Sense Media, 2023; Piper Sandler, 2025).

The rapid integration of smartphones into young people’s daily routines has raised increasing concern among parents, teachers, and policymakers about their possible effects on development. A growing amount of research indicates that digital technologies can have both positive and negative effects on youth. On the positive side, smartphones help facilitate communication with friends, enhance social networks, give access to educational resources, and promote opportunities for creativity and identity exploration (Valkenburg and Peter, 2011; Nesi, 2020). At the same time, an expanding body of research has raised worries about the potential consequences of heavy smartphone use. Studies have found links between high levels of digital media engagement and increased symptoms of anxiety and depression, sleep problems, attention fragmentation, and lower academic focus among teenagers (Orben and Przybylski, 2019; Twenge and Campbell, 2018; Paulus et al., 2023; Santos et al., 2023). While many of these effects seem small at the population level, researchers are increasingly pointing out that certain patterns of smartphone use, such as excessive social media activity, late-night use, and digital multitasking, can be especially relevant to youth well-being and learning outcomes.

These concerns have sparked renewed debate about appropriate boundaries for smartphone use among young people, especially in educational settings. One of the clearest policy responses has been the adoption of restrictions or bans on mobile phone use during school hours. In recent years, schools across the United States and around the world have put policies in place to limit smartphone access during instructional time to reduce digital distractions and boost classroom engagement (Beland and Murphy, 2016; Goodyear et al., 2025). Supporters of these policies argue that restricting smartphone use can help lessen attention disruptions and create better learning environments. Meanwhile, critics question whether restrictions during school hours effectively address broader patterns of digital engagement outside of school.

Interestingly, although many adults expect strong resistance from young people toward smartphone restrictions, emerging evidence suggests that adolescents themselves may have more nuanced views on technology limitations. Some studies reveal that after an initial adjustment period, students report better concentration and classroom engagement in environments where phone use is restricted. These findings raise important questions about youth perspectives on smartphone use and technology regulation. Understanding whether adolescents support certain types of smartphone restrictions, and under what conditions, can provide valuable insights into the potential effectiveness and acceptance of such policies.

### Study scope and research questions

The widespread adoption of smartphones among children and adolescents has generated a rapidly expanding literature examining their influence on cognitive development, psychological well-being, and educational outcomes. Although numerous empirical studies have investigated specific aspects of smartphone use, findings remain heterogeneous because digital engagement is not a single, uniform behavior. Instead, outcomes appear to depend on factors such as purpose of smartphone use, duration and timing of use, developmental stage, and individual vulnerability.

Accordingly, the purpose of this integrative, narrative review is to synthesize current evidence regarding the cognitive and psychological effects of smartphone use in children and adolescents. Furthermore, emerging policy responses designed to reduce digital distraction are examined. Throughout this review, children are defined as individuals between 8 and 12 years of age, while adolescents refer to youth approximately 10–19 years of age, consistent with the World Health Organization definition. Studies including older adolescents and emerging adults were included when they provided insight into developmental processes relevant to adolescent technology use.

This review addresses the following research questions:

1. What is currently known regarding he relationship netween smartphone use and psychological well-being, including sleep, mental health, and social functioning, in children and adolescents?
2. How does smartphone use influence attention, executive functioning, learning, and academic performance?
3. Which psychological and cognitive theories best explain observed relationships between smartphone use and developmental outcomes?
4. What evidence exists regarding smartphone restriction policies, including school cellphone bans and other phone-free environments, and how effective are these interventions?
5. What important methodological and conceptual gaps remain in the literature, and what directions should future research pursue?

The remainder of this review synthesizes empirical evidence addressing these questions while integrating theoretical perspectives from cognitive psychology, developmental psychology, educational research, and public health.

## Methods

This manuscript was conducted as an integrative narrative review. Unlike systematic reviews, which aim to identify and quantitatively synthesize all eligible studies using predefined inclusion criteria, integrative narrative reviews seek to synthesize evidence across diverse study designs to provide conceptual understanding of complex and multidisciplinary topics (Baumeister and Leary, 1997; Ferrari, 2015; Snyder, 2019). Because the cognitive and developmental effects of smartphone use span psychology, neuroscience, education, public health, and policy research, and integrative narrative approach was considered the most appropriate methodology.

Searches were performed using PubMed, PsycINFO, and Google Scholar to identify peer-reviewed articles examining the relationship between smartphone or screen media use and outcomes among children, adolescents, and young adults. Additional reports and policy analyses were identified through searches of institutional and government databases, including publications from organizations such as the Pew Research Center, the National Center for Education Statistics (NCES), and the Kaiser Family Foundation.

Search queries included combinations of keywords such as *smartphone use*, *screen time*, *digital media*, *adolescent mental health*, *well-being*, *sleep*, *academic performance*, *attention*, *digital distraction*, and *school cellphone bans*. Boolean operators (AND/OR) were used to refine search results and identify studies addressing specific outcomes such as mental health, sleep, cognitive performance, and academic outcomes.

Studies published between 2005 and 2025 were considered for inclusion in order to capture research conducted during the rapid expansion of smartphone ownership and social media use. Priority was given to peer-reviewed empirical studies, systematic reviews, and meta-analyses examining digital media use among youth populations. Additional policy reports and educational analyses were included where relevant to provide context regarding the implementation of school smartphone restrictions.

Articles were included if they examined at least one of the following outcomes:

1. Mental health or psychological well-being,
2. Sleep and health-related behaviors,
3. Cognitive functioning or academic performance, or
4. Policy interventions related to smartphone use in educational settings.

Studies focusing solely on unrelated forms of digital technology or adult-only populations were excluded unless they offered foundational theoretical frameworks related to digital distraction or cognitive load. Review of reference lists from key articles was also conducted to find additional relevant publications.

The final body of literature included experimental studies, observational studies, systematic reviews, and policy analyses, allowing for a comprehensive synthesis of research examining the potential benefits and risks associated with smartphone use among youth.

### Historical rise of Mobile technology use among children and adolescents

Over the past two decades, mobile technology has dramatically changed the daily lives of children and teenagers, with smartphones, tablets, and social media platforms becoming deeply embedded in social interactions, entertainment, and learning environments. Smartphone ownership in the United States grew rapidly during the 2010s, increasing from about 35% of adults in 2011 to over 90% by the mid-2020s (Pew Research Center, 2024). Among teenagers, access has become nearly universal; recent surveys estimate that roughly 95% of U.S. teens now have a smartphone (Pew Research Center, 2023). This widespread adoption marks a significant shift in the technological experience of young people.

**Figure 1:**
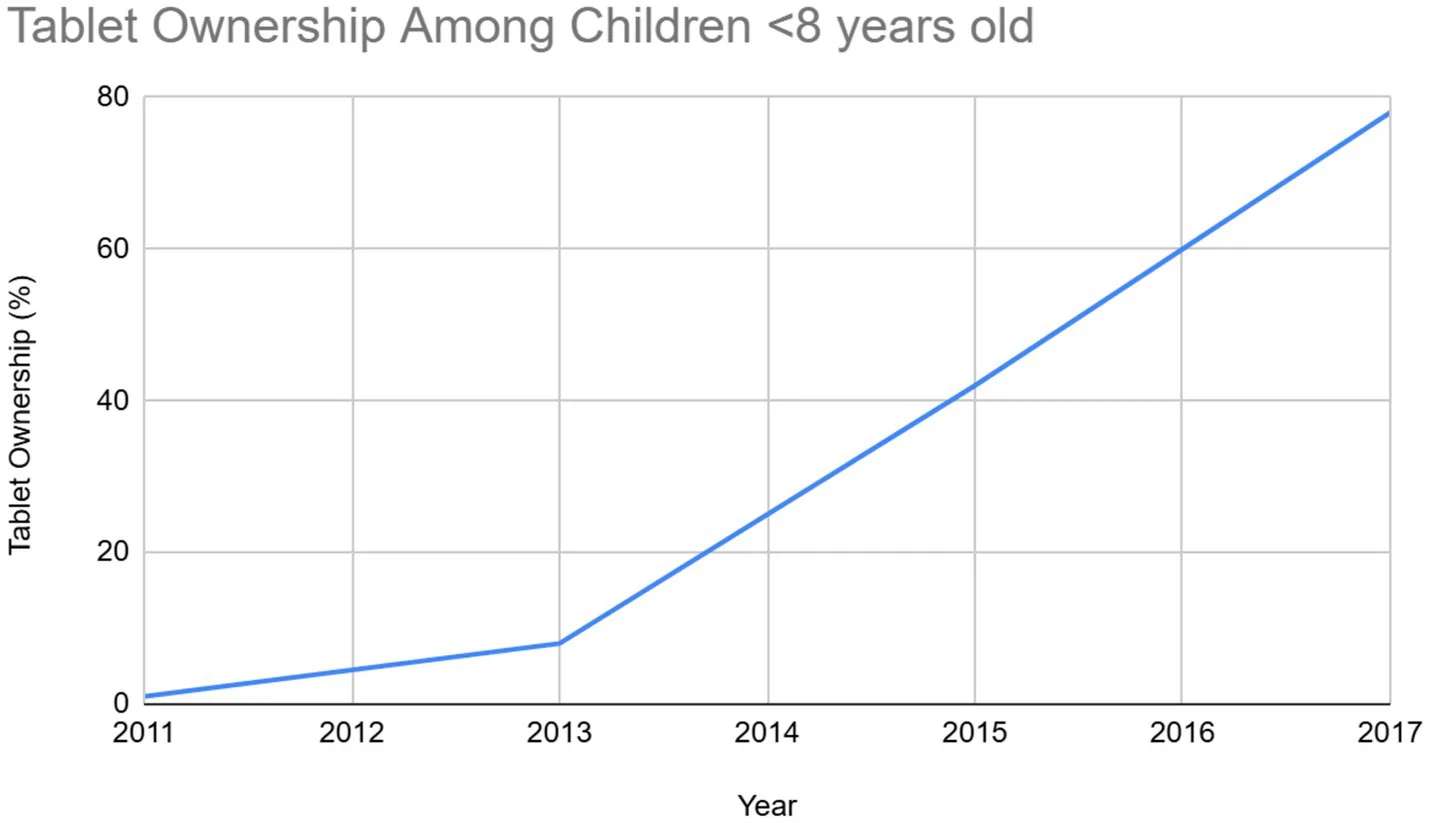
This line graph here shows a dramatic rise in tablet ownership among children under 8 years old, increasing from almost 0% in 2011 to almost 80% in 2017. This sharp upward trend shows how digital exposure begins increasingly earlier in childhood. Data obtained from Pew Research.

The expansion of digital access has also occurred at increasingly younger ages. Data from Common Sense Media (2025) indicate that ownership of mobile devices among children under the age of eight has increased dramatically over the past decade. As illustrated in Figure 1, tablet ownership among children under eight rose from nearly zero in 2011 to approximately 80% by 2017, highlighting how digital exposure is beginning earlier in childhood than in previous generations. The normalization of mobile devices at such young ages raises important developmental considerations, as screens increasingly mediate entertainment, communication, and early learning experiences.

**Figure 2:**
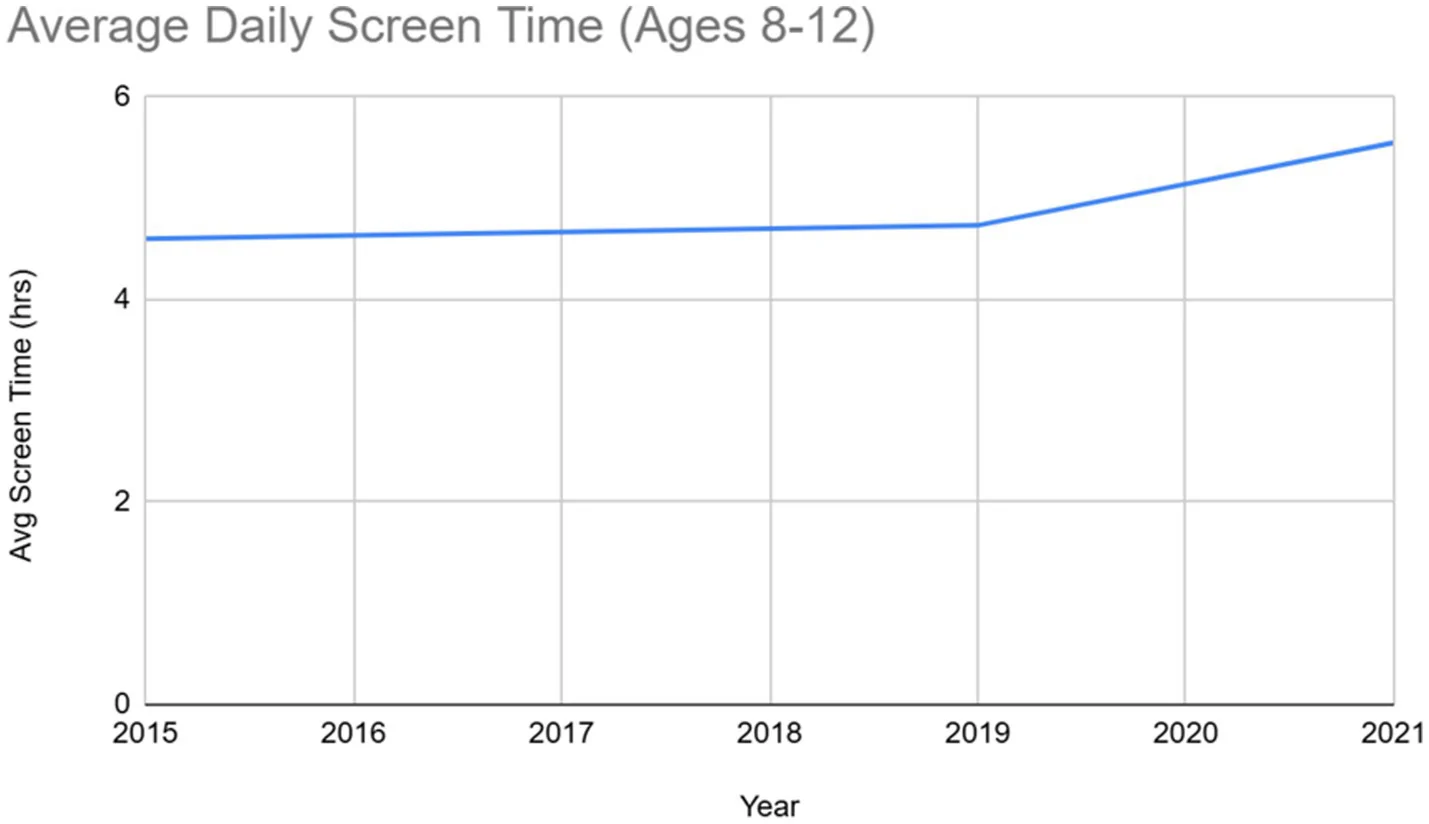
This figure displays a steady increase in daily screen time among 8–12 year olds, shooting up from 4.5 h per day to almost 6 h per day by 2021. The upward slope began even steeper around 2020, likely reflecting on the impacts of the COVID-19 pandemic. Data from Pew Research.

In addition to expanding access, the time children spend on screens has also increased significantly. Figure 2 shows a steady rise in daily screen time among children aged 8–12 years, from about 4.5 h per day to nearly 6 h per day by 2021. Notably, the increase seems to speed up around 2020, likely reflecting behavioral changes related to the COVID-19 pandemic, such as remote schooling, fewer outdoor activities, and greater dependence on digital media for social interaction. This upward trend indicates that screen-based activities have become deeply ingrained in children’s daily routines.

**Figure 3:**
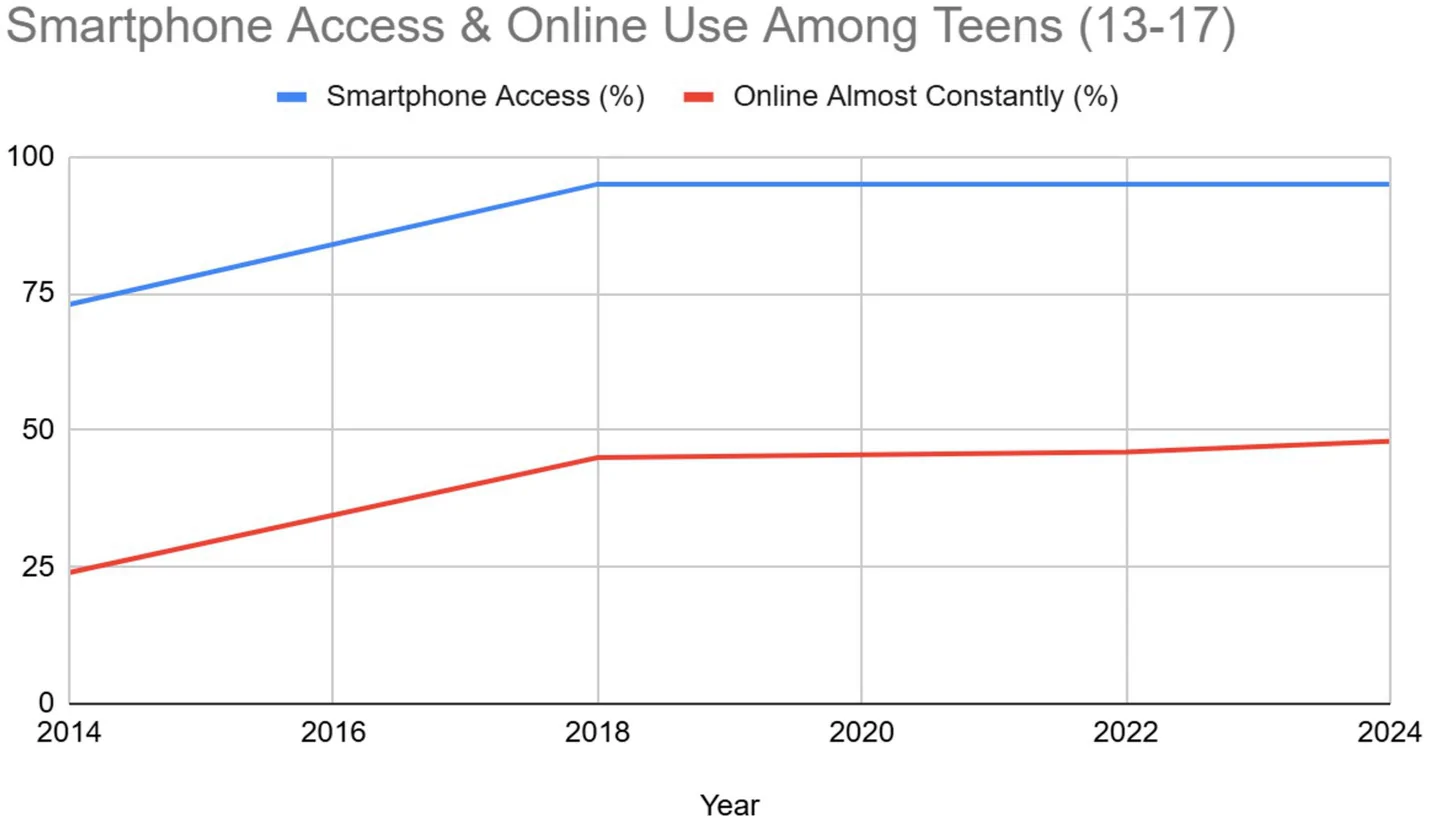
This dual-line graph illustrates two parallel trends: Increasing smartphone access and growing rates of teens who report being “online almost constantly.” While smartphone access has reached almost 95%, the percentage of teens who are online almost constantly has also had a significant climb. Data from Pew Research.

The link between access and behavior becomes even more evident during adolescence. As illustrated in Figure 3, smartphone access among teenagers has reached about 95%, while the share of teens saying they are “online almost constantly” has also risen significantly in recent years. These similar trends show that the issue is not just device ownership but also how often and intensely teens engage digitally. Constant connectivity may affect various behavioral and psychological outcomes, including sleep routines, attention control, and social comparison processes (Orben and Przybylski, 2019).

**Figure 4:**
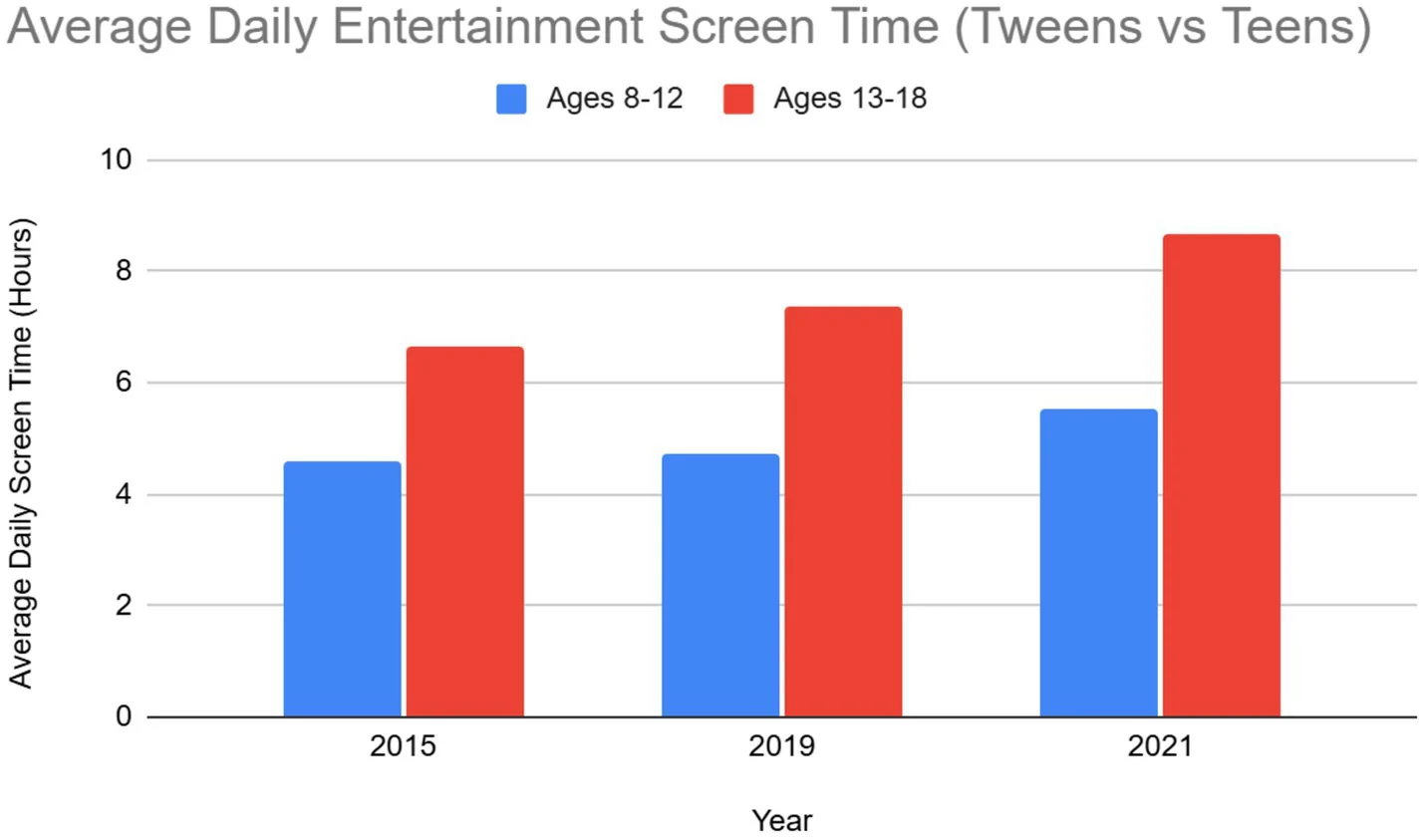
This bar chart compares screen time for entertainment purposes across two age groups—tweens (8–12) and teens (13–18). From 2015 to 2021, both groups show steady increases, with teens consistently having higher average times.

Patterns of digital media use also change as children grow older. As shown in Figure 4, entertainment screen time rises with age, with adolescents consistently reporting higher daily usage than younger children. From 2015 to 2021, both tweens (ages 8–12) and teens (ages 13–18) experienced steady increases in entertainment-related screen time, with adolescents showing the highest overall engagement. These results indicate that as children mature, digital involvement not only grows longer but also shifts more toward entertainment-based media consumption.

**Figure 5:**
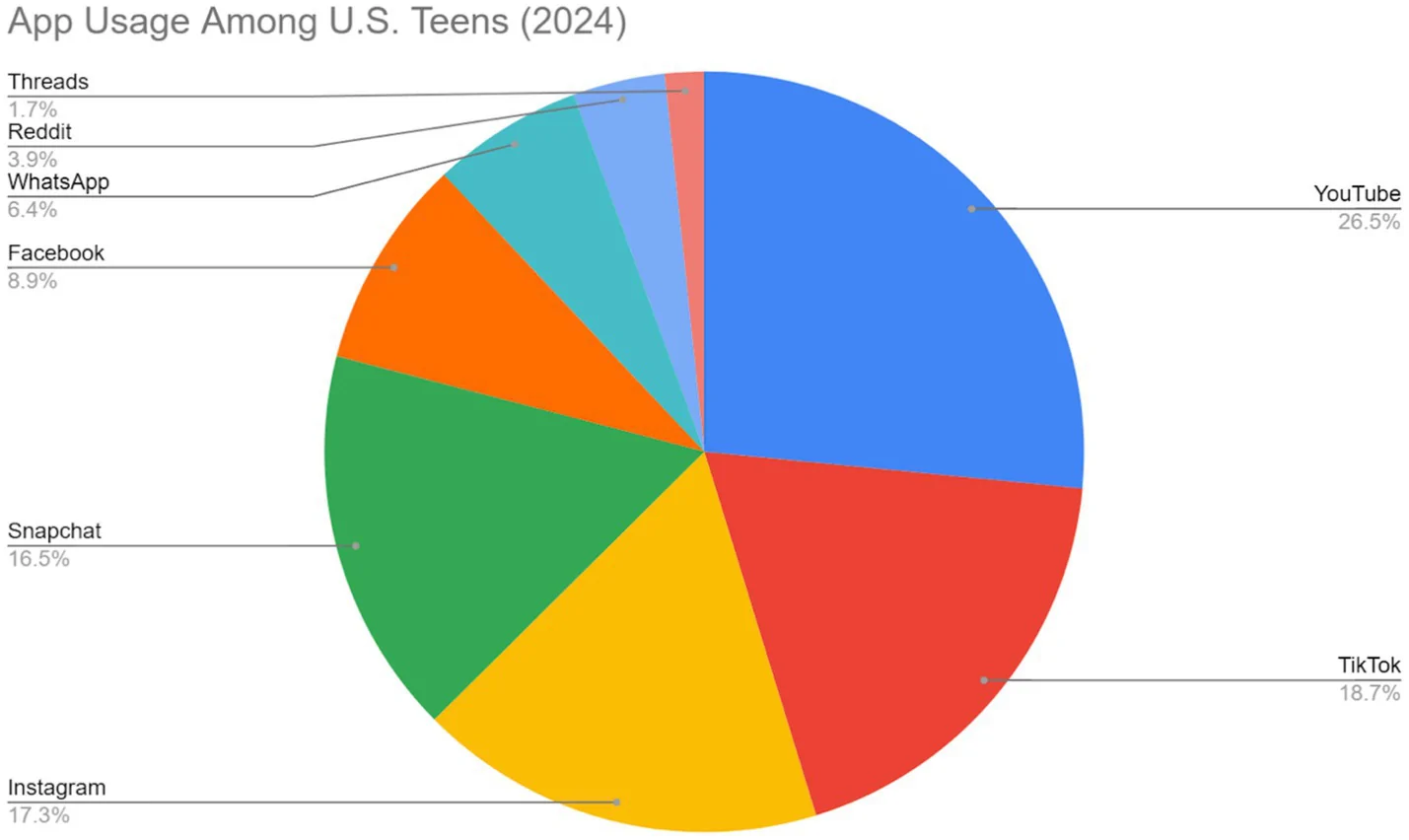
This pie chart breaks down which apps dominate U.S. teen’s attention in 2024. YouTube (26.5%), TikTok (18.7%), Instagram (17.3%), and Snapchat (16.5%) make up the vast majority, all platforms known for high engagement and algorithm-driven content.

The platforms capturing the most teen attention further reveal the nature of modern digital environments. Figure 5 shows the share of attention given to major social media and content platforms among U.S. teens. YouTube has the largest share of teen engagement (26.5%), followed by TikTok (18.7%), Instagram (17.3%), and Snapchat (16.5%). These platforms are marked by algorithm-driven content feeds and highly engaging short-form videos, designed to boost user interaction and encourage frequent use. In line with national survey data, YouTube remains the most popular platform among adolescents, while TikTok, Instagram, and Snapchat collectively form the core of today’s teen social media landscape (Pew Research Center, 2023; Piper Sandler, 2025).

Taken together, these trends show a rapid change in youth digital worlds. Mobile devices are now used earlier in childhood, screen time is rising across different developmental stages, and algorithm-driven social platforms lead adolescent media use. Understanding these historical changes helps explain how digital technology might affect young people’s cognitive, social, and emotional health.

### Psychological theories explaining the impact of smartphone use on youth development and well-being

No single psychological theory adequately explains the complex relationship between smartphone use and adolescent development. Rather, contemporary research suggests that smartphone-related outcomes emerge through multiple interacting cognitive, motivational, and social mechanisms. The theoretical frameworks reviewed below can therefore be organized into three complementary domains. First, cognitive theories explain how smartphones compete for limited attentional resources and influence learning. Second, social theories describe how digital communication alters interpersonal relationships and social comparison processes. Finally, motivational theories explain why adolescents are drawn to smartphones and why digital engagement can become habitual or excessive. Together, these perspectives provide an integrated framework for interpreting the empirical findings presented later in this review.

### Cognitive mechanisms

#### Distraction hypothesis

The distraction hypothesis proposes that smartphones disrupt cognitive functioning by pulling attention away from primary tasks (Kahneman, 1973). Human attention is limited, and when individuals try to split their focus between multiple stimuli, such as schoolwork and smartphone notifications, their performance on the main task often drops (Kahneman, 1973). Smartphones cause frequent interruptions from notifications, messages, and social media alerts, which can disrupt attention and make it harder to stay focused on mentally demanding activities.

Experimental research strongly supports this framework. Ward et al. (2017) showed that even when turned off, the mere presence of a smartphone can lower working memory capacity and impair reasoning task performance. Similarly, Stothart et al. (2015) found that receiving smartphone notifications can distract attention and reduce task performance, even when individuals do not actively respond to the alert. In educational settings, these attentional disruptions may lead to poorer learning outcomes. Studies indicate that students who use devices for non-academic purposes during lectures perform worse on exams and take fewer quality notes compared to students who stay focused on instruction (Ravizza et al., 2017; Kuznekoff and Titsworth, 2013).

Because adolescence is a crucial developmental period for executive functioning, including sustained attention, working memory, and self-control, frequent exposure to digital interruptions may influence cognitive habits and learning behaviors over time.

#### Cognitive load theory

Cognitive load theory provides another framework for understanding how smartphones can influence learning processes. According to this theory, the human working memory system has limited capacity, and learning happens most efficiently when cognitive resources are focused on relevant instructional material (Sweller, 1988).

When students try to multitask with academic activities and smartphone use, their cognitive resources become divided between competing tasks. Notifications, messages, and social media interactions can boost extraneous cognitive load, leaving fewer resources for processing and remembering new information.

Research examining classroom multitasking supports this idea. Students who use devices for non-academic purposes during lectures usually show lower comprehension and worse academic results (Ravizza et al., 2017). These findings indicate that digital multitasking can interfere with the deep thinking needed for learning and long-term memory creation.

Together, these theories suggest that smartphones influence cognition primarily by competing for finite attentional and working-memory resources. From this perspective, interruptions, multitasking, and constant accessibility increase extraneous cognitive load, reducing the cognitive resources available for learning, problem-solving, and sustained attention.

### Social mechanisms

Beyond cognitive effects, smartphones alter the social environments in which adolescents develop. Several theories propose that digital media influence well-being by changing how young people interact with others and evaluate themselves in social contexts.

#### Social displacement theory

Another widely discussed framework is social displacement theory, which suggests that time spent engaging with digital media may replace activities that support healthy psychological and social growth (Kraut et al., 1998). According to this view, increased use of smartphones might decrease the time adolescents spend in face-to-face interactions, physical activity, academic work, and sleep.

Research on adolescent media use aligns with this theory. Adolescents who report more recreational screen time tend to have less physical activity, poorer sleep, and feel less social support (Zablotsky et al., 2025). Systematic reviews also link increased screen time with higher levels of anxiety and depression among teens (Santos et al., 2023).

However, the displacement framework has become more nuanced in recent years. Smartphones also facilitate social interaction by allowing adolescents to maintain friendships, communicate with peers, and participate in online communities. As a result, researchers increasingly emphasize that the effects of digital media may depend on the type and context of technology use rather than simply the total amount of time spent online.

#### Displacement–stimulation framework

While early research often viewed digital media as replacing beneficial offline activities, recent scholarship has introduced the displacement–stimulation framework as a more nuanced explanation of how digital communication technologies impact social development and well-being. This framework combines elements of social displacement theory with evidence that digital communication can also promote social interaction.

The displacement perspective suggests that time spent using digital media may replace activities that contribute to healthy development, such as face-to-face social interaction, physical activity, academic work, and sleep (Kraut et al., 1998). In contrast, the stimulation perspective proposes that digital communication technologies can strengthen social relationships by increasing opportunities for interaction, maintaining friendships across distance, and facilitating access to supportive communities (Valkenburg and Peter, 2007).

Empirical studies suggest that both processes can happen at the same time. For example, adolescents who spend a lot of time passively consuming social media content might face social displacement effects, such as less in-person interaction and more feelings of loneliness. However, adolescents who mainly use digital platforms for communication, like messaging friends, joining group chats, or participating in supportive online communities, might experience stimulation effects that strengthen existing social ties.

Research examining adolescent online communication supports this dual-process model. Valkenburg and Peter (2007) found that online communication with existing friends was associated with increased closeness and friendship quality among adolescents. Similarly, studies of digital peer interaction suggest that social media can provide important opportunities for emotional support, particularly for youth who may feel socially marginalized in offline environments.

The displacement–stimulation framework suggests that the psychological effects of smartphone use depend mainly on how people engage with technology rather than how much they use it. Passive content consumption, excessive screen time, or using devices at night may lead to negative outcomes through displacement and attention problems. Conversely, active communication and positive online interactions can enhance social connection and well-being.

This perspective aligns with increasing evidence that digital media effects depend on context and vary among individuals. Adolescents who are already socially connected may use digital platforms to strengthen relationships, while those experiencing loneliness or social challenges might be more susceptible to negative comparisons or excessive screen time. Consequently, many researchers now focus on examining patterns of digital engagement, including activity type, timing, and social context, instead of only measuring total screen time.

Integrating the displacement–stimulation framework with other psychological theories offers a more complete understanding of how smartphones affect youth development. While attentional disruption, social comparison, and cognitive load may account for some negative outcomes, the stimulation perspective emphasizes how digital technologies can promote communication, identity exploration, and social support.

#### Social comparison theory

Social comparison theory offers another explanation for how social media may affect adolescent well-being. Proposed by Festinger (1954), the theory states that individuals assess their abilities, achievements, and social status by comparing themselves to others.

Social media platforms often showcase curated images of peers’ lives, including accomplishments, social events, and appearance-focused content. Viewing these idealized images can foster upward social comparisons, which may result in feelings of inadequacy, lower self-esteem, and heightened depressive symptoms (Nesi, 2020). Adolescents tend to be especially susceptible to these impacts because identity development and peer acceptance are key developmental tasks during this period.

Research suggests that passive social media use, like scrolling through content without interacting, may be especially linked to negative social comparison effects. On the other hand, active participation, such as messaging friends or joining supportive online communities, might lead to more positive social outcomes.

Collectively, these theories suggest that digital communication is not inherently beneficial or harmful. Rather, developmental outcomes depend upon whether smartphone use replaces beneficial offline experiences, strengthens existing relationships, or increases maladaptive social comparison.

### Motivational mechanisms

While cognitive and social theories explain many consequences of smartphone use, they do not explain why adolescents are so strongly motivated to engage with smartphones. Motivational theories address the psychological needs that digital media fulfill.

#### Uses and gratifications theory

While some theoretical perspectives highlight the potential harms of digital media, uses and gratifications theory emphasizes the motivations behind media use. According to this framework, individuals actively choose media platforms to fulfill specific psychological or social needs, such as entertainment, information seeking, social connection, and identity exploration (Katz et al., 1973).

Adolescents often use smartphones to keep up with friends, explore their interests, and find emotional support. Digital platforms also offer chances for creativity, teamwork, and getting information that can boost learning and personal growth. From this view, smartphone use is not naturally harmful; instead, its impact depends on how individuals incorporate digital media into their lives.

Research guided by uses and gratifications theory has shown that adolescents who use digital media mainly for communication and maintaining relationships often report more positive experiences than those who mostly passively consume content (Nesi, 2020).

#### Self-determination theory

Another key framework for understanding smartphone engagement is self-determination theory (SDT), which highlights the importance of intrinsic motivation and fundamental psychological needs in influencing behavior. According to SDT, people are motivated to participate in activities that fulfill three essential psychological needs: autonomy, competence, and relatedness (Deci and Ryan, 2000).

Digital platforms frequently offer instant feedback, social validation, and interactive opportunities that can fulfill these needs. For instance, social media “likes” and comments may boost feelings of competence and connection, while the ability to create and share content promotes autonomy and self-expression. However, when engagement turns into overuse or compulsive behavior, these same mechanisms might lead to problematic patterns of technology usage.

Some researchers contend that smartphone apps are intentionally created to exploit psychological reward mechanisms by offering intermittent reinforcement through notifications and social feedback. This setup might promote repeated checking behaviors and extended usage, especially among teenagers whose self-regulatory systems are still maturing.

These theories suggest that adolescents actively seek smartphones because digital environments satisfy fundamental psychological needs for autonomy, competence, relatedness, entertainment, and social connection. However, the same reward mechanisms may also contribure to excessive or compulsive use.

#### Integrating psychological frameworks

Although these theretical perspectives emphasize different mechanisms, they should not be viewed as competing explanations. Rather, they describe complementary processes operating simultaneously during smartphone use. For example, an adolescent checking social media while studying may experience attentional disruption predicted by the Distraction Hypothesis, increased extraneous cognitive load described by Cognitive Load Theory, reduced time devoted to academic work consistent with Social Displacement Theory, upward comparisons with peers predicted by Social Comparison Theory, and continued engagement driven by the motivational processes described in Uses and Gratifications Theory and Self-Determination Theory. Consequently, smartphone use should be understood as a multidimensional developmental exposure involving interacting cognitive, social, and motivational mechanisms rather than a single behavioral phenomenon.

The theoretical framework presented here provides the conceptual foundation for interpreting the empirical literature reviewed in the following sections. Importantly, it also suggests that the effects of smartphone use are likely to depend not only on the amount of smartphone use but also on the purpose, context, timing, and developmental stage of the user.

#### Smartphone use and youth well-being

The rapid integration of smartphones and digital media into the daily lives of children and adolescents has led to extensive research on their potential impacts on youth well-being. Overall, the literature indicates a complex relationship where the effects of smartphone use on mental health are generally modest but vary widely among individuals and situations. These studies are summarized in Table 1.

**Table 1:** Empirical studies examining smartphone use and youth well-being.

Study	Population/sample	Study design	Key outcomes	Main findings
Orben and Przybylski (2019)	Large datasets of adolescents (>350,000 participants across multiple surveys)	Secondary data analysis	Psychological well-being	Digital technology use showed small negative associations with well-being; effects were modest and heterogeneous.
Paulus et al. (2023)	Youth samples across multiple studies	Critical review	Mental health and neurodevelopment	Average mental health effects are small, but outcomes depend on sleep patterns, social context, and individual vulnerability.
Santos et al. (2023)	Adolescents across multiple countries	Systematic review	Anxiety, Depression	Consistent associations between higher screen time and internalizing symptoms; causality remains uncertain.
Dibben et al. (2023)	Adolescents in prospective cohort studies	Systematic review of longitudinal research	Sleep and mental health	Sleep disruption frequently mediates relationships between device use and poorer well-being; nighttime use particularly problematic.
Zablotsky et al. (2025)	U.S. teenagers (national dataset)	Cross-sectional analysis	Physical activity, sleep, mental health	≥4 h/day of non-school screen time associated with depression/anxiety symptoms, irregular sleep, lower physical activity, and lower social support.
Poulain et al. (2025)	Children and adolescents	Observational study	Quality of life	Problematic smartphone use associated with lower quality of life, with stronger associations observed during the pandemic period.
Schmidt-Persson et al. (2024)	Families with children and adolescents	Randomized clinical trial	Mental health, sedentary behavior	Two-week reduction in leisure screen time improved short-term mental health and prosocial behavior.
Carter et al. (2016)	Children and adolescents	Systematic review and meta-analysis	Sleep outcomes	Screen-based media device use linked to delayed sleep onset, shorter sleep duration, and poorer sleep quality.
Scott et al. (2019)	Adolescents	Observational study	Sleep patterns	Social media use associated with later bedtimes and shorter sleep duration.
Zink et al. (2024)	Late childhood	Longitudinal analysis	Sleep quality and insomnia	Bidirectional relationship between screen time and sleep disturbances; timing and type of digital activity are important.

Large-scale analyses show that digital technology use has small negative links with well-being measures at the population level (Orben and Przybylski, 2019). Likewise, recent reviews combining neuroscience and behavioral research find that although widespread concerns about digital media use are valid, overall mental health effects are generally small and vary greatly based on contextual and individual factors (Paulus et al., 2023).

Despite the modest size of these associations, systematic reviews consistently report links between higher levels of screen engagement and increased internalizing symptoms among adolescents. A systematic review of studies examining screen time and adolescent mental health found consistent links between greater total screen exposure and symptoms of anxiety and depression, although the strength of these relationships varied across studies and causal pathways remain uncertain (Santos et al., 2023). These findings indicate that while screen use alone is unlikely to be the sole cause of youth mental health outcomes, it may interact with other behavioral and environmental factors that affect adolescent well-being.

One of the main ways smartphone use affects well-being is through sleep disruption. Teens often use smartphones in the evening, and exposure to screens before bed has been linked to later sleep onset, shorter sleep duration, and poorer sleep quality (Cain and Gradisar, 2010). Prospective studies show that sleep problems often explain the connection between electronic device use and mental health outcomes (Dibben et al., 2023). Newer research also indicates that the link between screen time and sleep may go both ways. Increased screen time, especially late at night, has been associated with worse sleep quality and insomnia symptoms, while teens with sleep problems may be more likely to use digital media at night (Chen and Lyu, 2024). Physiological research further suggests that using smartphones in the evening and overnight may disrupt circadian rhythms and reduce sleep duration, which can affect mood regulation and cognitive performance (LeBourgeois et al., 2017).

Besides sleep-related factors, various behavioral and lifestyle habits can influence the link between smartphone use and well-being. Studies with U.S. adolescents indicate that spending four or more hours per day outside of schoolwork or recreational screen time is linked to several negative health outcomes. These include decreased physical activity, disturbed sleep patterns, heightened body image concerns, increased depressive and anxiety symptoms, and less perceived social support (Zablotsky et al., 2025). On the other hand, research with college students suggests that regular physical activity may act as a protective factor, with higher exercise levels connected to less problematic smartphone use or “addiction” (Pirwani and Szabo, 2025). These results emphasize the need to consider overall lifestyle habits when assessing the impacts of digital media use.

Another emerging research area focuses specifically on this problematic smartphone use. Recent studies show increases in problematic smartphone behaviors among children and adolescents, along with declines in self-reported quality of life measures. In particular, the links between problematic smartphone use and lower quality of life seem to have grown stronger in recent years, especially during the COVID-19 pandemic and post-pandemic period when digital engagement rose significantly (Poulain et al., 2025). While the cause-and-effect relationship remains hard to determine, these findings suggest that excessive or uncontrolled smartphone use may be a risk factor for reduced well-being among certain groups of youth.

Experimental research on interventions offers more insight into the potential link between screen use and well-being. A randomized clinical trial examining the effects of reducing recreational screen time found that a two-week, family-based reduction in leisure screen use resulted in decreased sedentary behavior and short-term improvements in children’s mental health and prosocial behavior (Hansen et al., 2024). While these findings were limited to short-term outcomes, they suggest that changing digital media habits may provide measurable benefits for youth well-being.

Importantly, recent policy-focused analyses highlight that smartphone use and adolescent mental health should not be viewed only through the lens of school settings. Evidence from the SMART Schools study and related research indicates that merely restricting phone use during school hours is unlikely to significantly improve mental health outcomes. Instead, many of the mechanisms connecting digital media use to well-being, such as sleep disruption, social comparison, and cyberbullying, mainly take place outside of school environments (Goodyear et al., 2025). Consequently, researchers increasingly emphasize the need for broader strategies that combine school policies with family-based approaches, digital literacy education, and targeted support for youth who are particularly vulnerable to problematic technology use.

Taken together, the current literature suggests that the relationship between smartphone use and youth well-being is multifaceted and context-dependent. While average population-level effects appear modest, certain patterns of digital engagement, such as excessive screen time, nighttime device use, and problematic smartphone behaviors, may increase the risk of sleep disruption, reduced physical activity, and poorer mental health outcomes. At the same time, smartphones can also facilitate social connection and access to information, highlighting the importance of examining how, when, and why adolescents use digital technologies rather than focusing solely on total screen time.

#### Digital media use and academic performance

Beyond potential effects on emotional well-being, a growing body of research has explored how smartphone use may impact cognitive functioning and academic performance. A subset of these studies, described in the narrative of this section, are summarized in Table 2. Much of this research concentrates on how digital devices introduce attentional disruptions that can interfere with learning processes. Smartphones offer constant access to notifications, messaging platforms, and social media apps, all competing for limited cognitive resources. From a cognitive standpoint, these interruptions may add extra extraneous cognitive load, reducing the mental resources available for processing instructional material (Sweller, 1988).

**Table 2:** Empirical studies examining smartphone use, attention, and academic performance.

Study	Population/sample	Study design	Outcome measures	Key findings
Ward et al. (2017)	University students	Laboratory experiments	Working memory, fluid intelligence	The mere presence of a smartphone reduced cognitive capacity, even when the phone was turned off or silenced.
Stothart et al. (2015)	Adult participants	Experimental study	Attention task performance	Phone notifications alone disrupted attention and impaired performance, even without interacting with the device.
Ravizza et al., 2017	College students	Classroom observational study	Exam performance	Students engaging in off-task internet use during lectures scored lower on exams than students who remained focused on course material.
Kuznekoff and Titsworth (2013)	College students	Experimental lecture study	Note-taking quality and recall	Students who texted during lectures demonstrated reduced note quality and poorer information recall.
Junco (2012)	University students	Survey and academic record analysis	GPA and academic engagement	Frequent social media use during academic activities associated with lower academic engagement and GPA.
Aagaard (2015)	University students	Qualitative study	Student perceptions of distraction	Students reported frequent technology-driven distractions during lectures and difficulty self-regulating device use.
Chen et al. (2016)	Students	Observational research	Academic performance	Problematic smartphone use associated with lower academic achievement and increased distraction.
Beland and Murphy (2016)	Secondary school students	Natural experiment (school phone bans)	Standardized test scores	Schools implementing phone bans saw improvements in test scores, particularly among lower-performing students.

Experimental studies provide strong evidence that smartphones can influence cognitive performance even when they are not actively being used. In a series of laboratory experiments, Ward et al. (2017) demonstrated that the mere presence of an individual’s smartphone placed on a desk or in a nearby bag reduced performance on tasks measuring working memory and fluid intelligence. Importantly, these effects occurred even when the device was turned off or silenced, suggesting that the cognitive demands associated with resisting smartphone-related thoughts may consume attentional resources. These findings support the idea that smartphones can impose a form of background cognitive load, subtly diminishing the mental capacity available.

Related research shows that smartphone notifications can interrupt attention. Stothart et al. (2015) discovered that receiving a phone notification, even when participants did not interact with the device, caused noticeable declines in performance on tasks that require focus. The size of these disruptions was similar to the effects seen when people actively used their phones, indicating that even quick digital interruptions can break concentration and hurt cognitive performance.

Research conducted in educational environments provides further evidence linking distractions from smartphones to academic results. Classroom studies have shown that students who use technology for non-academic purposes during lectures tend to perform worse on assessments than those who stay focused on the instruction (Damiao and Cavaliere, 2021). For instance, (Ravizza et al., 2017) found that students who used laptops or smartphones for unrelated internet activities during lectures had significantly lower exam scores compared to peers who avoided off-task digital behavior. Similarly, Kuznekoff and Titsworth (2013) reported that students who texted during lectures exhibited poorer note-taking quality and understanding of the course material.

These findings support research on the wider phenomenon of digital multitasking. When students try to engage with academic materials and smartphone-based communication or social media at the same time, their cognitive resources are divided between competing tasks. This type of multitasking has been linked to less thorough processing, weaker memory encoding, and poorer learning outcomes (Junco, 2012). Qualitative research also indicates that students often underestimate how much off-task technology use impacts their attention, highlighting the difficulty of self-regulating digital distractions in learning environments (Aagaard, 2015).

Overall, the evidence indicates that smartphones may affect cognitive functioning and learning mainly through mechanisms involving attentional interference and cognitive load. While digital technologies also offer valuable educational tools and access to information, excessive smartphone use during instructional activities can decrease students’ ability to maintain attention and engage in deep learning. Understanding how smartphones influence attentional environments in educational settings is therefore essential for creating strategies that promote both effective learning and responsible technology use.

The growing body of research connecting smartphone use to attentional problems, sleep issues, and potential effects on adolescent well-being has led educators and policymakers to consider structural measures aimed at reducing digital distractions in schools. Specifically, concerns about multitasking during lessons, declining classroom engagement, and the impact of social media on student focus have encouraged many schools to introduce policies that restrict or ban mobile phone use during the day. While psychological theories like the distraction hypothesis and cognitive load theory suggest that limiting access to phones during instruction may enhance learning outcomes, the evidence supporting these policies is mixed. Some studies indicate that restricting smartphone use in classrooms can boost student attention and academic results, but others show that these limits have minimal effect on broader well-being because many digital activities happen outside school. The next section reviews recent research on school cell phone bans and assesses how these policies affect classroom learning, student behavior, and overall adolescent well-being.

#### School cell phone bans and policy responses

Concerns about how smartphone use might affect students’ attention, academic success, and well-being have led to more policies aimed at restricting mobile phone access in schools. Recently, school districts across the U.S. and around the world have adopted various rules that limit or ban student smartphone use during classes or the entire school day. These efforts show a growing understanding among educators and policymakers that smartphones can serve as digital distractions that hinder learning.

##### National trends in school phone restrictions

At the national level, restrictions on mobile phone use in schools are becoming more common. Recent federal education data show that about 77% of U.S. public schools ban cell phone use during instructional time, with roughly 30% extending these restrictions beyond class periods to limit device use throughout the school day (National Center for Education Statistics, 2025). Similarly, policy analyses indicate that around three-quarters of U.S. public schools now restrict or ban phone use during class, reflecting a broad shift toward device management strategies in educational settings (Kaiser Family Foundation, 2025).

Public opinion seems to support these policy changes. National survey data show that about 74% of U.S. adults favor banning smartphones in middle and high schools, reflecting a growing belief that limiting device access during school hours can help students focus and improve learning outcomes (Pew Research Center, 2025). Education policy reports also indicate that mobile phone bans are spreading quickly across school districts and states, as educators increasingly note that student smartphone use leads to classroom distraction and lower engagement (Education Week, 2025a,b).

##### State-level policy initiatives

Beyond local school policies, several U.S. states are enacting broader legislative measures to control smartphone use in schools (Education Week, 2023). Recent policy updates show that at least 15 states have passed laws or guidelines at the state level that limit mobile phone use during school hours, often in response to concerns from educators about classroom disruptions and decreasing student attention (National Education Association, 2024). For instance, policies in states like New York, Florida, and California have set rules requiring students to store their phones during class time or the entire school day.

Some states have adopted even stricter laws. In Ohio, lawmakers recently passed a law banning student cell phone use during school hours, citing the need to cut distractions and boost academic focus (Dayton Daily News, 2024). Likewise, New York has launched a statewide program requiring students to store their phones during the school day as part of a broader “distraction-free schools” initiative. Early reports from teachers in districts with such policies indicate improvements in classroom focus, participation, and overall student engagement (Rockefeller Institute of Government, 2024).

##### International policy approaches

School smartphone restrictions are not exclusive to the United States. Several countries have adopted national or regional policies to limit student phone use during the school day. France was among the first to implement a nationwide ban on smartphones in middle schools, requiring students to keep devices stored during school hours. Evaluations of these policies have reported improvements in student social behavior and decreases in bullying incidents after implementation. More recently, France expanded these restrictions through pilot programs requiring students to lock away their phones during the entire school day, with early feedback indicating improvements in classroom focus and peer interaction.

Other countries have adopted similar strategies. Finland began restricting mobile phone use during lessons in 2025, with early reports indicating that classrooms became calmer and students showed better concentration. In the United Kingdom, research on school phone bans found that limiting smartphone access was linked to better student test scores, especially among lower-achieving students who are more susceptible to classroom distractions.

### Evidence on the effects of phone bans

Despite the rapid growth of school phone restrictions, research on their effectiveness shows mixed results. A quick review of smartphone bans in European schools found that these policies were linked to improvements in social well-being, such as fewer student conflicts and better classroom social climates. However, evidence on academic performance was more limited, with some studies reporting small test score gains and others showing minimal effects.

Similarly, qualitative research on student experiences with phone bans indicates that such policies can enhance perceived academic focus and social interactions when combined with broader strategies that promote digital literacy and student well-being. However, other studies suggest that school-based restrictions may have limited impact on overall patterns of smartphone use. These studies are summarized in Table 3.

**Table 3:** Empirical evidence on school cell phone ban policies and educational outcomes.

Study	Country/context	Study design	Policy interventions	Key outcomes	Main findings
Beland and Murphy (2016)	United Kingdom secondary schools	Natural experiment using administrative data	School-wide mobile phone ban	Standardized test scores	Phone bans were associated with improved student test scores, with the strongest effects observed among lower-performing students.
Kessel et al., (2019)	Sweden secondary schools	Observational policy evaluation	School-level smartphone restrictions	Academic performance	Schools implementing phone restrictions reported improvements in student concentration and academic outcomes, though results varied across schools.
Goodyear et al. (2025)	United Kingdom schools (SMART Schools study)	Cross-sectional observational study	Schools with varying phone restriction policies	Mental health, phone use	No significant differences in mental health or well-being were observed between schools with strict vs. permissive phone policies.
OECD education policy reports	Multiple countries	Policy Analysis	Classroom smartphone restrictions	Learning environment	Many schools report reduced classroom distractions following phone restrictions, though causal evidence remains limited.
Common Sense Media reports	United States	Survey research	School phone policies	Student perceptions	Students often report improved concentration and social interaction after an adjustment period following phone restrictions.
Lancet Regional Health policy analysis	Europe	Policy commentary and synthesis	School phone bans	Youth mental health	Evidence suggests school phone bans alone are insufficient to address broader mental health concerns, as key mechanisms occur outside school environments.

One of the largest recent studies examining school phone policies, the SMART Schools study, found that schools with restrictive phone policies did not show significant differences in student mental health, sleep patterns, attention, or behavioral outcomes compared to schools with more permissive policies (Goodyear et al., 2025). Although restrictive policies reduced smartphone use during school hours, they did not significantly decrease overall social media engagement or device use outside school settings. These findings suggest that while school phone bans may improve classroom environments, they might not substantially impact broader well-being outcomes without additional strategies targeting technology use outside of school.

Overall, current research indicates that the link between smartphone use and youth well-being is complicated and influenced by many factors. Instead of only looking at total screen time, future studies and policies might gain from examining usage patterns, contextual details like sleep schedules, and individual differences in susceptibility. Understanding how these factors work together is crucial for creating evidence-based approaches that promote healthy digital habits among children and teenagers.

### Student perspectives on smartphone restrictions

Understanding student perspectives on smartphone policies is also crucial for assessing how effective these interventions are. Surveys that explore youth attitudes toward technology restrictions indicate that students often have nuanced views about smartphone use in school environments (Nisar, 2020; RAND Corporation, 2024; Screenagers, 2024; The Guardian, 2025; UK Parliament Lords Library, 2024). According to the fall 2025 Pew Research report, 41% of teens 13–17 supported banning cellphone use during class (Pew Research Center, 2025). However, only 17% support bans for the entire school day (Pew Research Center, 2025). Although many teachers say that smartphones are a major source of distraction in the classroom, students themselves frequently underestimate how much their phone use disrupts learning (Education Week, 2025a,b).

Simultaneously, research shows that students can be greatly influenced by their peers’ phone use. Surveys reveal that over half of students feel distracted when others use phones during class, emphasizing the collective effect of digital disruptions in learning settings (OECD, 2023). Additionally, studies indicate that adolescents spend large parts of their day on smartphones both during and outside school hours, with some estimates suggesting that teens may dedicate up to a quarter of their school day to using mobile devices (Seattle Children’s Research Institute, 2024). According to a study by Christakis et al., students average 1.5 h on their phones out of an average 6.5 h school day, and 1 in 4 students report over 2 h of use (Christakis et al., 2025).

In a large-scale study performed in South Australia, Bar et al. found that students report both positive and negative effects of cell phone bans (Bar et al., 2025). Students report that having access to their phones during school promotes emotional support and reduced anxiety, a sense of independence and trustworthiness, and assistance with learning (Bar et al., 2025). In contrast, students recognize that school-day access to phones contributes to decreased academic engagement, including increased distraction and decreased motivation (Bar et al., 2025). However, there are benefits of bans from a student perspective. Interactions with peers increased, academic performance improved, and benefits to both mental and physical well-being were noted (Bar et al., 2025; Böttger and Zierer, 2024).

More broadly, attitudes among generations toward smartphones are becoming more ambivalent. Surveys of Generation Z young adults indicate that a significant minority have concerns about the impact of digital technologies in their lives. For instance, one national survey found that about one in five Gen Z respondents wished smartphones had never been invented, while another poll revealed that nearly half shared similar feelings about platforms like TikTok and related technologies (Harris Poll, 2024; Asia Financial, 2024). These results suggest that young people themselves may see both the advantages and challenges of constant digital connectivity.

### Smartphone restrictions in extracurricular and recreational settings

While much of the existing research on smartphone restrictions has focused on school environments, similar policies are increasingly being adopted in extracurricular and recreational settings. Residential summer camps, in particular, often implement phone-free policies aimed at promoting social interaction, outdoor activities, and participation in camp events. Surveys of North American camps show that most maintain formal guidelines limiting digital media use, with some camps banning smartphones entirely during the entire program (DeHudy, 2018). These policies are often presented as opportunities for adolescents to experience temporary “digital detox” environments that decrease technological distractions and foster face-to-face communication.

Emerging qualitative research suggests that phone-free environments can significantly affect adolescents’ social experiences. For example, interviews with teenagers at smartphone-free residential camps show that participants often report stronger peer connections and greater involvement in group activities when mobile devices are not allowed (Megret, 2023). Similarly, early studies on digital detox experiences at summer camps indicate that temporarily removing smartphones may lower stress, enhance focus on activities, and promote healthier sleep patterns among teens (Reed and Gies, 2026). These results support broader research suggesting that continuous digital connection can interfere with face-to-face social interactions and sustained attention in youth settings.

In high school sports, teens also report positive effects of cell phone bans during practices and other team events (Comstock and Fioresi, 2026). The Sierra Vista High School girls’ basketball team implemented a cell phone ban, which reduced player distraction, improved player bonding, and, according to their coach, resulted in a dramatic improvement in their season, resulting in a state playoff appearance (Comstock and Fioresi, 2026).

Despite the widespread implementation of phone restrictions in camps and youth programs, however, empirical research examining their effects remains limited. Most rigorous studies evaluating smartphone bans have focused on school-based policies and outcomes such as classroom attention and academic performance. In contrast, relatively little research has explored how temporary phone-free environments outside school influence adolescents’ broader digital habits, including potential rebound effects in screen time, changes in peer interaction patterns, or longer-term attitudes toward smartphone use. Because youth spend substantial portions of their time in extracurricular and recreational settings, understanding how technology restrictions function in these environments represents an important area for future research.

### Implications of the research

Despite rising concerns about youth smartphone use, evidence suggests that simple restrictions, like bans on phones at school, may not be enough to enhance adolescent well-being on their own. Analyses of school phone policies show that many factors affecting mental health, such as sleep disruption, cyberbullying, and social comparison, happen outside school environments (Weiss and Bonell, 2025).

Therefore, effective interventions may need to include broader strategies that involve families, digital literacy education, and community-based support networks. Encouragingly, experimental research shows that behavioral interventions can lead to measurable improvements. A randomized clinical trial where families reduced leisure screen time for 2 weeks resulted in better mental health and prosocial behavior in children, along with less sedentary time (Schmidt-Persson et al., 2024). Although long-term outcomes are still uncertain, these findings suggest that reducing screen exposure intentionally can have a positive impact on youth well-being.

Consequently, many scholars support more comprehensive strategies that integrate school policies with digital literacy education, family technology guidelines, and targeted support for adolescents who might be especially vulnerable to problematic smartphone use. These approaches can create a more balanced framework for managing smartphone use while maintaining the benefits digital technologies offer for communication, learning, and social connection.

### Research gaps and future directions

Despite the quickly growing research on smartphone use and youth development, several key gaps still exist in the literature. One major challenge is the difficulty of establishing cause-and-effect links between smartphone use and mental health outcomes. Much of the current research relies on cross-sectional or correlational methods, which make it hard to tell whether smartphone use leads to declines in well-being or if adolescents with psychological issues are more likely to use digital media more. Although some longitudinal and experimental studies have started to explore this, more research with prospective designs, randomized interventions, and natural experiments will be necessary to clarify the direction of these relationships.

Another significant limitation of the current literature is the heavy reliance on self-reported measures of screen time and smartphone use. Self-reports are often prone to recall bias and may not accurately capture the timing, duration, or type of digital activities adolescents participate in. Future research would benefit from including objective digital usage data, such as passive smartphone sensing or application-level usage logs, which can offer more accurate insights into how specific types of digital engagement relate to developmental outcomes.

A third area needing more research involves the diversity of smartphone use behaviors. Much of the initial research on digital media focused on total screen time; however, increasing evidence indicates that the effects of smartphone use largely depend on the type of activity, the social setting, and the timing of device use. For instance, active communication with peers may enhance social bonds, while passive scrolling through social media content might heighten social comparison and feelings of loneliness. Likewise, using a smartphone in the evening or at night seems especially linked to sleep disturbances. Future studies should therefore go beyond simple measures of screen time to explore patterns of digital engagement, including specific platforms, social interactions, and timing of usage.

The literature also emphasizes the need for more research on individual differences in vulnerability and resilience. Not all adolescents seem equally affected by digital media use. Factors such as pre-existing mental health conditions, social support networks, personality traits, and family environments may influence the connection between smartphone use and well-being. Knowing which groups of youth are most at risk for problematic technology use will be crucial for creating targeted interventions and support systems.

Finally, there remains limited evidence regarding the long-term effectiveness of policy interventions, such as school smartphone bans. While some studies suggest that restricting phone use during instructional time may improve academic outcomes or classroom engagement, evidence regarding broader impacts on mental health and overall technology habits remains mixed. Many of the mechanisms linking smartphone use to well-being occur outside school environments. As a result, future research should explore comprehensive approaches that combine school policies with family-based strategies, digital literacy education, and community-level interventions designed to promote balanced and healthy technology use.

Taken together, these research gaps highlight the need for interdisciplinary approaches integrating perspectives from psychology, neuroscience, education, and public health. As smartphones continue to evolve and become further embedded in everyday life, developing a more nuanced understanding of how digital technologies influence youth development will be essential for informing evidence-based policies and practices that support adolescent well-being.

### Implications for educators, parents, and policymakers

The findings discussed in this article have significant implications for educators, families, and policymakers who want to promote healthy technology use among children and adolescents. First, research on attention, cognitive load, and classroom distractions indicates that reducing non-academic smartphone use during lessons can improve the learning environment. Schools might benefit from establishing clear policies that limit device use during class while still permitting purposeful educational technology applications. Organized strategies, such as device storage systems, designated technology-use times, or digital etiquette rules, can help minimize distractions while preserving the benefits of digital tools for learning.

Second, research highlighting the role of sleep disruption in mediating the relationship between smartphone use and well-being suggests that families should consider strategies to limit nighttime device use. Encouraging adolescents to keep smartphones outside the bedroom at night or establishing “digital curfews” may help improve sleep quality and reduce late-night social media engagement. Because sleep plays a central role in emotional regulation, cognitive functioning, and physical health, promoting healthy digital habits around bedtime may have broad developmental benefits.

Third, the research indicates that the quality and context of digital engagement can be more important than total screen time alone. Educators and parents might benefit from helping adolescents develop digital literacy skills that promote more intentional use of technology. This could include teaching young people to recognize algorithm-driven content patterns, understand how social comparison impacts them on social media, and create strategies for managing notifications and digital distractions.

For policymakers, the mixed evidence regarding school cell phone bans highlights the importance of adopting balanced approaches to technology regulation. While restricting phone use during school hours may reduce classroom distractions, broader strategies may be necessary to address the full range of factors influencing adolescent well-being. Policies that promote digital education, support research on technology use among youth, and encourage collaboration between schools, families, and technology companies may be particularly valuable.

Ultimately, encouraging healthy technology use involves recognizing both the benefits and possible challenges of digital media. Smartphones and online platforms can enhance communication, creativity, and access to information, but they also raise new developmental concerns related to attention, sleep, and social comparison. By adopting balanced, developmentally appropriate strategies for technology use, educators, parents, and policymakers can help ensure that digital tools support, rather than hinder, the well-being and learning of young people.

## Conclusion

The rapid integration of smartphones and digital media into the lives of children and teenagers marks one of the most important technological changes affecting modern growth. The literature reviewed in this article suggests that the link between smartphone use and youth well-being is complicated and shaped by many interacting factors, such as usage patterns, developmental stages, and social environments. Psychological frameworks like the distraction hypothesis, social displacement theory, social comparison theory, uses and gratifications theory, self-determination theory, and cognitive load theory offer useful insights into how digital technologies can impact attention, social behavior, and emotional health. Empirical studies show that while the overall effects of smartphone use on mental health are generally small, certain usage patterns are linked to sleep problems, attention issues, and lower academic achievement. These patterns include excessive screen time, nighttime device use, and multitasking during learning.

At the same time, smartphones offer significant benefits, including opportunities for communication, social support, and access to educational resources. Therefore, the impact of digital media on youth development cannot be understood solely in terms of harm or benefit. Instead, outcomes largely depend on how and when technology is used, as well as individual differences in vulnerability and resilience. This complexity is also reflected in policy responses such as school cell phone bans. While these policies may reduce classroom distractions and boost student engagement in some situations, current evidence indicates that restricting phone use during school hours alone may not significantly affect broader patterns of technology use or adolescent well-being.

Future research should therefore go beyond simple measures of screen time and instead focus on the quality, context, and timing of digital engagement. Long-term studies examining how smartphone use interacts with development, sleep patterns, academic behaviors, and social relationships will be especially important. Additionally, interdisciplinary research combining insights from psychology, neuroscience, education, and public health may help clarify when digital technologies support healthy development and when they might pose risks. As mobile technologies continue to evolve, creating evidence-based strategies that promote balanced and intentional technology use will be crucial for supporting the well-being and learning of children and adolescents in an increasingly connected world.
